# Exploration of *Sarcoptes scabiei* Antigenic Protein Which Play Roles in Scabies Pathogenesis in Goats and Rabbits

**Published:** 2018

**Authors:** Nunuk Dyah Retno LASTUTI, Poedji HASTUTIEK, Lucia Tri SUWANTI, Dony CHRISMANTO

**Affiliations:** 1. Dept. of Veterinary Parasitology, Faculty of Veterinary Medicine, Universitas Airlangga, Surabaya, Indonesia; 2. Dept. of Animal Health, Faculty of Vocational, Universitas Airlangga, Surabaya, Indonesia

**Keywords:** *Sarcoptes scabiei* var. *caprae*, *Sarcoptes scabiei* var. *cuniculi*, Mites, Antigenic proteins

## Abstract

**Background:**

Scabies or mange is an infectious skin disease caused by the mite *Sarcoptes scabiei*. This skin disease affects various livestock such as goats, sheep, swine, cattle, other animals like dogs, cats, wild animals and also affect human. This research aimed to explore the protein in mites *S. scabiei* which has antigenic character and play roles in scabies pathogenesis in goats and rabbits.

**Methods:**

*S.scabiei* mites were isolated from goats and rabbits, and characterized using SDS-PAGE. In addition the protein was also analysed using Western Blot assay. The isolation and identification were carried out in 2015 at the Parasitology Laboratory of Veterinary Medicine Faculty, Universitas Airlangga, Surabaya, Indonesia.

**Results:**

The identification results using SDS-PAGE of mites *S. scabiei* var. *caprae* expressed 12 protein bands between 26,7 kDa and 205,8 kDa, continued by Western Blot showed 3 protein bands, after being reacted with blood serum from scabies infected goat, it could be identified antigenic protein with molecule weight 205.8 kDa, 57.3 kDa, and 43 kDa. While protein in mites *S. scabiei* var. *cuniculi* identified 9 protein bands between 24 kDa and 75 kDa by SDS-PAGE, and the Western Blot assay identified antigenic protein with molecule weight 62 kDa and 51 kDa.

**Conclusion:**

The antigenic protein of *S. scabiei* var. *caprae* and *S. scabiei* var. *cuniculi* showed that they are probably involved in the scabies pathogenesis in goats and rabbits.

## Introduction

Scabies or mange is an infectious skin disease caused by the mite *Sarcoptes scabiei* and considered as one of important diseases in human and animal. It is estimated at more than 300 million people are infected each year (1). Sarcoptes mite infestation was reported attacking more than 100 mammal species including human and domestic animal (2). Nowadays, it is considered an emerging/re-emerging parasitic disease that threatens human and animal health globally (3). Sarcoptes mite is an obligate parasite, which develops in the skin, penetrates stratum corneum and forms burrow in order to complete the life cycle starting from the egg to the adult stage (4).

On domestic animals, pigs and goats seem to be the most susceptible, whereas, in pet animals, the disease is most common in dogs. In Japan, mange outbreaks were observed in raccoon dogs with high morbidity and mortality, and the debilitated animals caused by *S. scabiei* were often sent to veterinary hospitals and wildlife rescue facilities (5). Scabies is an endemic disease, but occasionally outbreaks can be occurred and attack most of cattle.

Sarcoptic mange is one of the most economically important diseases in goats in Indonesia, although treatment with drugs is generally effective for control of scabies and relatively high cost. Scabies occurrence on goats in Indonesia is between less than 5% to approximately 100% and mortality is quite high, the range is between 67%–100% on young goats and on adult goats is about 11% (6, 7). Due to the prevalence of the disease in human and animal are very high, the economic losses caused by the disease are enormous.

Phenomenon shows that difference variety of *S. scabiei* has different characteristic of specific antigenic protein or different immune-dominant, as *S. scabiei* var. *canis* isolated from dogs, contains immunogenic protein with molecule weight of 200, 185, 170, 155, 142,133, 112, 97, 74, 57, 45, 42, 32 and 22 kDa (8). Mites *S. scabiei* contain specific protein with molecule weight between 15 kDa and 225 kDa which based on protein identification results using SDS-PAGE (9), it was identified 33 bands where 18 bands are recognized by specific *S. scabiei* antibody using Western Blot. The research results of mites on dogs showed the strongest protein which causes allergy has molecule weight more than 90 kDa (10).

The purpose of the study was to explore the protein in mites *S. scabiei* which has antigenic character and play roles in scabies pathogenesis in goats and rabbits. The exploration of antigenic protein will provide information regarding the protein profile of *S. scabiei* of domestic goat and rabbit in Indonesia. They may also help to characterize and identify of immunogenic proteins and genetic profile of *S. scabiei* used preliminary study for development of sub-unit vaccine as alternative for scabies prevention on goats and rabbits in Indonesia.

## Materials and Methods

### Isolation and identification of S. scabiei mites from goats and rabbits

This study was approved by Ethical Committee, Faculty of Veterinary Medicine, Universitas Airlangga, No: 036-KE).

*S. scabiei* mites were isolated from domestic goats and rabbits that showed clinical signs of scabies, such as thickening of the skin, crust formation, alopecia on the area around eyes, ears, mouth, and legs. The isolation and identification were carried out in 2015 at Parasitology Laboratory of Veterinary Medicine Faculty, Universitas Airlangga, Surabaya, Indonesia. The area of the skin that has crust, was scrapped, and mites were put on object glass and given drops of 10% KOH, then mites were observed under light microscope using magnification of 40 times. *S.scabiei* identification based on key identification from Soulsby (11). After identification, mites isolation was required for protein characterizing by these following steps: mites (around one thousand mites) was put into petri dish and mixed with Phosphate Buffer Saline (PBS) solution and strained to get the result free of skin crust. Next, the result was washed with PBS solution and centrifuged at 3000 rpm for 10 min. Washing process was performed at least three times, in order to get mites free of dirty materials that carried from scraping process. The deposit mites would be formed as pellets would be kept in freezer at minus 80 °C, to be processed into homogenate protein (12). The pellets (mites) with homogenising medium:100 mM Tris-HCL buffer pH 7.4, 100 mM NaCl, 5 mM EDTA, 5 mM MgCl_2_ (13), was sonicated at 30 kHz and this step was repeated for 16 times, every sonication steps last for 4 min with break time for 2 min. The sonication result solution was centrifuged at 16.000 rpm for 5 min, the protein concentration of the resulting supernatant was measured using visible spectrophotometer with 595 nm wavelength.

### Whole protein characterization using SDS-PAGE

The gel (10 wells) was loaded with 8 μl of protein solution. The gel was allowed to run at 125 volt, 40 mA. The gel was stained Silver Nitrat (Bio-Rad, Singapore) to reveal the protein bands followed by destaining with the ionized water (13).

### Antigenic protein characterization using Western Blot.

The gel as the result from electrophoresis process of *S. scabiei* protein using SDS-PAGE, continued by Western Blot. The procedure of Western Blot was performed according to existing protocols (13). The antigenic protein of *S. scabiei* was detected by SDS-PAGE and transferred to a nitrocellulose membrane for 1 h in an electrophoretic transfer cell (Bio-Rad, USA).

## Results

**Fig. 1:**
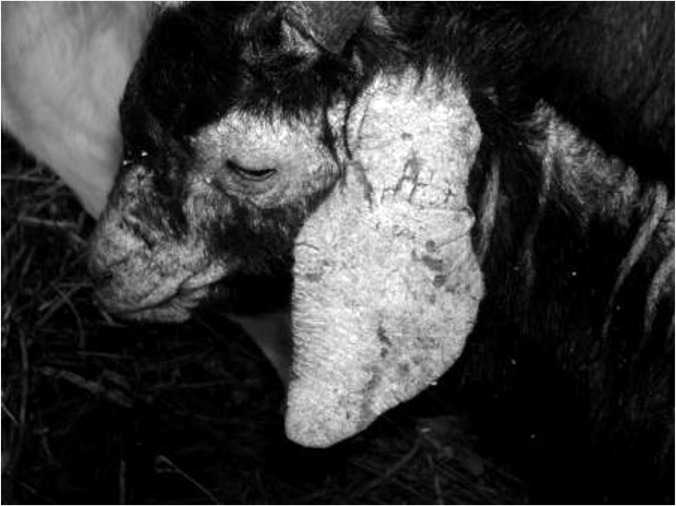
Goat with severe sarcoptic mange

Mites *S. scabiei* was isolated from goats and rabbits which show clinical signs of scabies such as thickening of the skin, crust formation, alopecia, erythematous papules on the area around eyes, ears, neck, back, muzzle. The photo was taken by Canon Ixus digital camera, Japan) (Fig. 1).

**Fig. 2:**
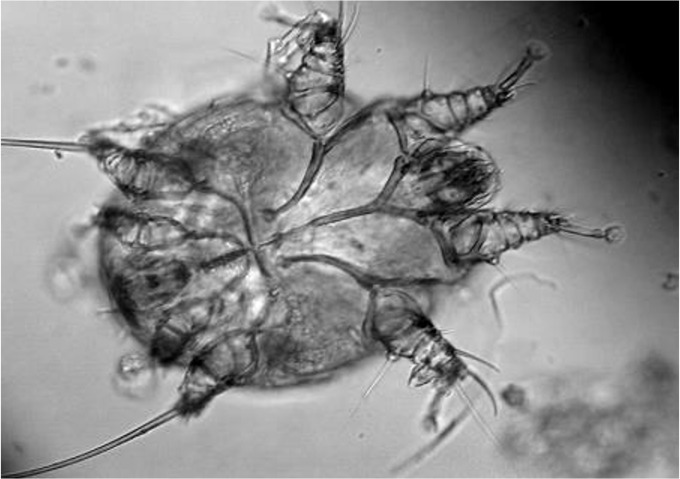
*Sarcoptes scabiei* ventral view of male, scraping from rabbit (magnification 400×)

The scraping result was put on object glass and given drops of 10% KOH, then mites were observed under light microscope Nikon using magnification of 400 times. Morphology of *S. scabiei* is a minute parasite, roughly circular in outline, adult female is approximately 300 to 500 μ long by 250 μ wide, and the male is slightly smaller, around 250 μ long by 200 μ wide. The sucker-bearing pairs legs have unjointed pedicels. All the legs of both sexes are short and the third and fourth pairs do not project beyond the margin of the body and are only visible from ventral view (Fig. 2).

**Fig. 3:**
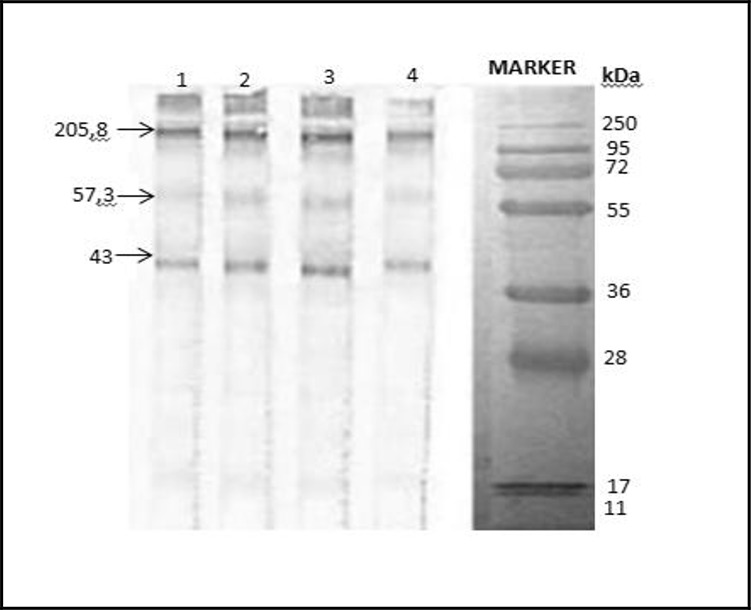
Western blotting analysis of S. scabiei var.caprae proteins. M, protein marker; lane1–4, protein of S. scabiei var, caprae

The characterization results of mites *S. scabiei* var. *caprae* protein by SDS-PAGE analysis 12% had identified 12 protein bands with molecule weight between 26.3 kDa and 205.8 kDa. According to the scanning results from SDS-PAGE analysis before performed Western blot analysis, it showed six kinds of protein were expressed, specifically protein 205.8 kDa, 187.4 kDa, 78.3 kDa, 57.3 kDa, 43 kDa and 40 kDa. The Western blot assay towards four out of six blood serum samples from goats with severe chronic stage as shown in Fig. 1, had identified three protein bands after being reacted with goat blood serum and added conjugate IgG anti-goat. Expressed protein had molecule weight 20.8 kDa, 57.3 kDa, 43.0 kDa, this three protein has antigenic characteristic which probably be involved in scabies pathogenesis on goats (Fig. 3).

**Fig. 4:**
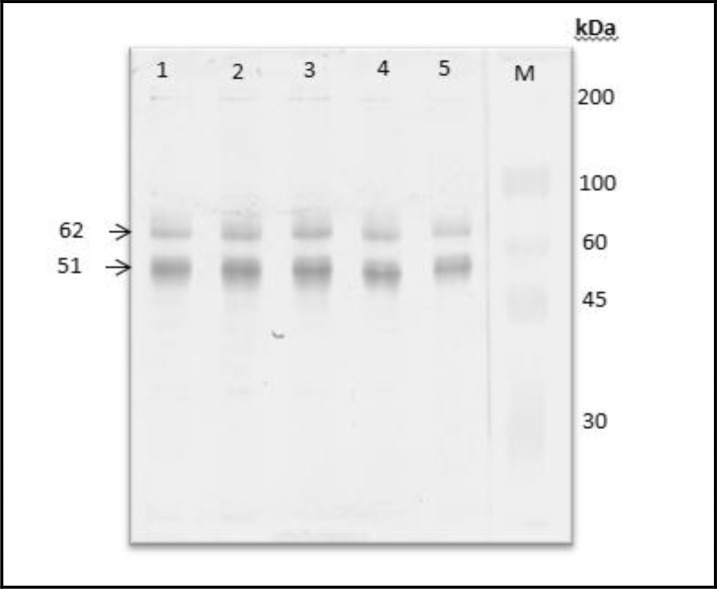
Western blotting analysis of S.scabiei var.cuniculi proteins. M, protein marker; lane 1–5, protein of S. scabiei var. cuniculi

Electrophoresis results of *S. scabiei* var. *cuniculi* protein isolated from four rabbits with severe scabies symptoms such as, alopecia, thickening of the skin and crust formation on the area around ears, eyes, nose and legs, was characterized by SDS-PAGE 12% and had identified nine protein bands with molecule weight between 24 kDa and 75 kDa. While characterization results by Western blot analysis had identified specific or antigenic protein using primer antibody from scabies infected rabbits serum and secondary antibody from conjugate IgG anti-rabbit, it expressed two antigenic protein bands specifically 62 kDa and 51 kDa (Fig. 4).

## Discussion

Based on the Western Blot analysis for determining antigenic protein which plays role in scabies pathogenesis, it showed the antigenic protein profile difference between *S. scabiei* var. *caprae* from goats and *S. scabiei* var. *cuniculi* from rabbits. For Western Blot analysis, it was used antigenic protein of *S. scabiei* reacted with primer antibody from scabies infected goats and rabbits serum. Pathogenesis of scabies disease is related to the host immune response after invaded by mites *S. scabiei*, it will penetrate the skin until reach the stratum corneum, then suck the lymph fluid and consume epidermis cells for its survival (4). Sarcoptes mite sucks the lymph fluid by damaging epidermis layer and consuming new epidermis tissue cells which cause irritation, continuous itch, and also causes wound. Skin will become erythema and form papule, vesicle and finally, inflammation reaction will occur followed by exudate formation. Exudate will settle at the skin surface so that crusts are formed and skin thickening will occur as well as hair loss (14).

The mechanism triggers clinical signs appearance is still not clearly defined, apparently it is related to hypersensitivity reaction type I, III and IV in human (15), and allegedly *S. scabiei* mites produce substance which activates type 1 T cells to produce IL-10 as anti-inflammation and immune-suppressive (3, 16). Mites protein is known as antigen, when antigen enters the body it will activate lymphocyte B cells to produce immunoglobulin, and also will activate Antigen Presenting Cell (APC) to present the peptide together with Major Histocompatibility Complex (MHC) which will be recognized by T Cells Receptor (TCR), the next process is differentiation and proliferation of T helper cells (Th) to become Th1 and Th2, Th2 will produce IL-4 which induces proliferation of lymphocyte B cells to produce immunoglobulin (IgG) (12, 18). Th 1 cells and Th2 cells are acted as immune response pattern, it also has been reported that Th2 cells produce IL-4 which stimulate lymphocyte B cells to produce IgE for increasing allergy immune response in scabies infected patient (16, 19).

For Western blot analysis, it was used primer antibody originated from naturally scabies infected goat antibody serum in Lamongan sub-district, East Java, the infected goats showed various clinical signs from mild to severe. Western Blot analysis results showed that there are three kinds of antigenic protein which play role in scabies pathogenesis. Those three protein have molecule weight as much as 205.8 kDa, 57.3 kDa, and 43.0 kDa. Protein with molecule weight 57.3 kDa has the highest content as much as 58.57% which measured by HPLC scan. It showed the characteristic of homogeny protein, and indicated that protein profile has stable antigenicity characteristic which can induce antibody both humoral and cellular, probably could be used development of vaccine (12, 29).

Specific antibody (primer antibody from scabies infected goats antibody serum and secondary antibody which is conjugate IgG anti-goat) can recognize specific antigen of *S. scabiei* var. *caprae* with molecule weight 205.8 kDa; 57.3 kDa; and 43.0 kDa. While antigenic protein of *S. scabiei* var. *cuniculi* with molecule weight 62 kDa and 51 kDa had been recognized by specific antibody (IgG) produced by rabbits that infected by scabies in the fields.

The antigenic protein profile difference is related to epitope difference which is the specific part of macromolecule antigen that binds antibody, in this case, epitope is a part of peptide that binds MHC molecule for being recognized by T cells receptors (17, 20–22). In a correlation with this terminology, antibody (IgG) produced by both scabies infected goats and rabbits are capable of binding epitope from *S. scabiei* antigen and expressed as protein bands with specific molecule weight. Antibody which produced by both scabies infected goats and rabbits is important components in immune system functioned in helping animal against particular antigen invasion. Antibody is produced in responding foreign antigen, and specific antibody (membrane receptors of lymphocyte B cells surface) will bind at specific antigen side (determinant antigen) which forms complex of antigen-antibody (20, 23).

Protein profile which has both antigenic and immunogenic character also has been expressed by various animal species; towards mites, *S. scabiei* var. *canis* isolated from red fox contains antigenic protein with molecule weight 15 kDa- 225 kDa (9). It has been investigated in identical animal (dogs) showing that mites *S. scabiei* var. *canis* contains antigenic protein with molecule weight 200, 185, 170, 155, 142,133, 112, 97, 74, 57, 45, 42, 32 and 22 kDa (8). In 2004, it had been isolated *S. scabiei* var. *caprae* mites, originated from goats in West Java, showing protein with molecule weight 43 to 220 kDa and recognized by specific scabies goats IgG, and the proteins with molecule weight 180, 135, 60, 43 and 38 kDa are very prominent and recognizable at day 10, it has been recognized by antibody IgE with molecule weight 130, 72, 64, 58, 44, 41, 39, 27 and 25 kDa (7).

The protein profile difference could be occurred due to: genotype variety (host species) difference (8, 24, 25). The result of mRNA sequencing from *S. scabiei* type *hominis* in locus DQ146410 showed length 766 bp (26), while the result of mRNA sequencing from *S. scabiei* isolated from goats (*caprae*) showed polypeptide with length 361 bp (27). On the development progress, both antigenic and immunogenic protein from *Sarcoptes scabiei* mites has been made recombinant for developing vaccine candidate as the choice for controlling scabies (15, 22, 28, 29).

## Conclusion

The protein molecule weight difference between mites *S. scabiei* var. *caprae* and *S. scabiei* var. *cuniculi* showed the difference between proteins which have specific antigenic character probably involved in the scabies pathogenesis in goats and rabbits. Further studies can be developed characterization and identification of immunogenic protein profile for subunit vaccine development.
